# Therapeutic Potential of Invariant Natural Killer T Cells in Autoimmunity

**DOI:** 10.3389/fimmu.2018.00519

**Published:** 2018-03-13

**Authors:** Luc Van Kaer, Lan Wu

**Affiliations:** ^1^Department of Pathology, Microbiology and Immunology, Vanderbilt University School of Medicine, Nashville, TN, United States

**Keywords:** invariant natural killer T cells, CD1d, immunotherapy, autoimmunity, type 1 diabetes, multiple sclerosis, rheumatoid arthritis, systemic lupus erythematosus

## Abstract

Tolerance against self-antigens is regulated by a variety of cell types with immunoregulatory properties, such as CD1d-restricted invariant natural killer T (iNKT) cells. In many experimental models of autoimmunity, iNKT cells promote self-tolerance and protect against autoimmunity. These findings are supported by studies with patients suffering from autoimmune diseases. Based on these studies, the therapeutic potential of iNKT cells in autoimmunity has been explored. Many of these studies have been performed with the potent iNKT cell agonist KRN7000 or its structural variants. These findings have generated promising results in several autoimmune diseases, although mechanisms by which iNKT cells modulate autoimmunity remain incompletely understood. Here, we will review these preclinical studies and discuss the prospects for translating their findings to patients suffering from autoimmune diseases.

## Introduction

Autoimmunity develops when tolerance against self-tissues becomes compromised. Such a breakdown of the normal mechanisms that promote self-tolerance may be triggered in a genetically susceptible host by environmental factors. Traditional treatments for autoimmune diseases have predominantly relied on immunosuppressive and anti-inflammatory agents that often only provide short-term relief. A more enticing outcome of immunotherapy would be to prevent clinical manifestations or to stop disease progression after its initiation. One potential way to accomplish this would be to re-establish tolerance by targeting immunoregulatory cell networks. A lot of the work in this field has centered on CD4^+^Foxp3^+^ regulatory T cells (Tregs), whereas other studies have concentrated on invariant natural killer T (iNKT) cells, a subset of glycolipid-reactive T cells. Here, we review the preclinical studies with iNKT cell antigens in mouse models of autoimmune disease, discuss proposed mechanisms for their therapeutic efficacy, and consider the hurdles faced in translating these findings to patients with autoimmune diseases.

## A Brief Primer on iNKT Cells and Their Functions

Invariant natural killer T cells express a semi-invariant T cell receptor (TCR), Vα14-Jα18/Vβ8.2, -7, or -2 in mice or Vα24-Jα18/Vβ11 in humans, and multiple surface markers associated with activated/memory T cells or natural killer (NK) cells [reviewed in Ref. (1–5)]. The semi-invariant TCR of iNKT cells recognizes glycolipid antigens presented in the context of the MHC class I-related protein CD1d. Relevant antigens for iNKT cells include both exogenous and endogenous glycolipids, many of which are glycosphingolipids. A common antigen employed in the iNKT cell field is the α-galactosylceramide (α-GalCer) KRN7000, which is a synthetically optimized version of a glycolipid originally isolated from a marine sponge (6).

In mice, functional subsets of iNKT cells, called iNKT1, iNKT2, iNKT10, and iNKT17 cells, have been identified that are characterized by production of the signature cytokines IFN-γ, IL-4, IL-10, and IL-17, respectively (7–11). These subsets are generated in the thymus, are characterized by expression of signature transcription factors, and are enriched in specific tissues, i.e., liver and spleen for iNKT1 cells; lungs and intestine for iNKT2 cells; adipose tissue and spleen for iNKT10 cells; and lungs, skin, and lymph nodes for iNKT17 cells. Additionally, a subset of iNKT cells that produces IL-21 and is specialized to interact with B cells to regulate humoral immunity (called iNKT_FH_ cells) has also been identified (12). In humans, similar subsets of iNKT cells expressing select transcription factors and cytokines have not yet been fully characterized.

Within hours of lipid antigen activation, iNKT cells can mount an effector response characterized by cytokine production and cytotoxicity (1–5). These cells therefore represent a critical component of the innate arm of the immune response. Activation of iNKT cells in this way also leads to the transactivation a variety of other immune cell types (13, 14). Consequently, iNKT cells can either promote or suppress immune responses in different diseases (15). They promote natural immunity to cancer, protect the host against some infections, typically suppress autoimmunity, and contribute to the development of a variety of inflammatory diseases (1–5, 15, 16). In both mice and humans that are predisposed to the development of autoimmunity, iNKT cells often are reduced in number and exhibit an IFN-γ-biased cytokine production profile (16–18), providing indirect evidence for a role of these cells in curbing autoimmunity.

Considering their immunoregulatory functions, the therapeutic activities of iNKT cells in disease have been examined (16, 17). These studies have provided evidence for therapeutic efficacy against tumors, infectious agents, and autoimmune and inflammatory diseases.

## *In Vivo* Response of iNKT Cells to Glycolipid Antigens

Most studies investigating the *in vivo* response of iNKT cells to glycolipid antigen activation have employed KRN7000, which when injected by the intraperitoneal route in mice, results in systemic iNKT cell activation (19, 20). Activation of iNKT cells in this way results in the following series of events: (a) KRN7000 is presented to iNKT cells by CD1d-expressing antigen-presenting cells, predominantly CD8α^+^ dendritic cells (DCs) (21). (b) iNKT cells become activated within hours, resulting in the induction of activation markers such as CD25, CD69, and ICOS. (c) iNKT cells rapidly but transiently produce cytokines, with an initial burst of IL-4 (1–8 h), followed by IFN-γ (12–36 h activation) (16). (d) These cells transiently (between 8 and 30 h after treatment) downregulate their TCRs (22). (e) They also downregulate surface expression of the NK cell marker NK1.1, which occurs as early as 24 h after treatment and can last for an extended time period (over 1 month) (22). (f) iNKT cells upregulate expression of the programmed death-1 (PD-1) inhibitory receptor, which is evident as early as 2–3 days after KRN7000 treatment and may last for an extended time period (up to 2 months) (23–25). (g) iNKT cells rapidly expand in multiple tissues (spleen, peripheral blood, bone marrow, and liver), which peaks around 3 days after treatment (22, 26). (h) The iNKT cell population returns to pre-treatment levels within 2–3 weeks, which is mediated by activation-induced cell death (22, 26, 27). (i) While iNKT cells lack classical immunological memory, these cells exhibit long-term alterations in immune responsiveness following lipid antigen stimulation. Specifically, *in vivo*-activated iNKT cells acquire a hyporesponsive or anergic phenotype, resulting in reduced proliferation and IFN-γ production in response to glycolipid antigen restimulation (27, 28). Such hyporesponsiveness was noted as early as 3 days until up to 2 months after KRN7000 treatment. Repeated intraperitoneal injection of KRN7000 is particularly powerful in inducing long-term iNKT cell anergy. While the physiological significance of this response remains uncertain, it may prevent persistent cytokine production in order to avoid chronic inflammation during situations where glycolipids are present for an extended time period (28). Mechanistic studies revealed a role for the PD-1/PD-L pathway in this process (23–25, 29, 30). It was also noted that these hyporesponsive iNKT cells exhibit regulatory properties due to their capacity to produce residual amounts of IL-4 (28) and increased levels of IL-10 (10), thereby suggesting that they might have adopted a phenotype characteristic of iNKT10 cells.

Administration of KRN7000 *via* the intraperitoneal or intravenous routes predominately activates iNKT1 and to a lesser extent iNKT2 cells in spleen and liver, but does not activate iNKT2 cells in lymph nodes (9). However, oral administration of KRN7000 stimulates iNKT2 cells in mesenteric lymph nodes (9). The latter manner of administration also avoids induction of iNKT cell anergy (31), as does administration *via* the intradermal (32) and intranasal (31) routes, in the context of strong co-stimulation (28, 33), blockade of the PD-1/PD-L pathway (23, 24, 34), nanoparticles (35), or recombinant CD1d molecules (36). Due to differences in the distribution of tissue-specific iNKT cell subsets, different mouse strains induce divergent responses to KRN7000, with BALB/c mice generating IL-4-biased iNKT cell responses and SJL/J mice generating IFN-γ-biased responses as compared with C57BL/6 mice (9, 37).

Although information is limited, studies with human subjects have shown that KRN7000 and related glycolipids can promote iNKT cell cytokine production and expansion (38). Additionally, repeated KRN7000 treatment caused progressively lower iNKT cell responses in these patients (39), thereby suggesting anergy induction. When KRN7000 was delivered to patients pre-loaded on DCs, such iNKT cell dysfunction was avoided (40).

The cytokine production profile of iNKT cells can be modulated by a variety of means, such as the strength and quality of co-stimulation, the presence of cytokines, as well as the nature of the glycolipid antigen employed (16, 41, 42). Structural variants of KRN7000 have been identified that deviate iNKT cell responses toward T helper (Th)1 or Th2 cytokine production (16, 41, 42), or that fail to induce iNKT cell anergy (43). These methods to modulate iNKT cell cytokine responses have been exploited for the development of improved iNKT cell-based therapeutics.

## Impact of iNKT Cell Antigens on Innate and Adaptive Immune Responses

Invariant natural killer T cells are engaged in extensive crosstalk with other immune cell types, which greatly impacts their therapeutic activities (16). Glycolipid-activated iNKT cells activate and enhance cytokine production by DCs and macrophages, modulate the functions of neutrophils, and influence the generation, recruitment, and functions of myeloid-derived suppressor cells (MDSCs). Glycolipid-activated iNKT cells also induce IFN-γ production and cytotoxicity in NK cells (44). iNKT cell antigens also influence adaptive immune responses, including CD8 and CD4 T cell responses, as well as B cell and antibody responses. Most evidence suggests that KRN7000 enhances Th2 immunity, especially when administered repeatedly (16, 45, 46). Structural variants of KRN7000 that further bias adaptive immune responses toward Th2 cytokine production (e.g., OCH and C20:2) or that instead promote Th1 immunity (e.g., α-C-GalCer) have been identified (16). Additionally, iNKT cell antigens can enhance the generation and suppressive properties of CD4^+^Foxp3^+^ Tregs (16, 47). These effects of glycolipid-activated iNKT cells on immune responses formed the scientific premise for investigating the therapeutic activities of KRN7000 and related glycolipids in a variety of diseases, including autoimmune diseases.

## Preclinical Studies of iNKT Cell Antigens in Autoimmunity

The immunomodulatory activities of KRN7000 and related iNKT cell antigens have been investigated in mouse models of autoimmunity (16, 18, 48). Key studies in select autoimmune diseases are reviewed here and potential mechanisms will be discussed in the next section.

### Autoimmune Diabetes

Several research groups have evaluated the effects of KRN7000 in non-obese diabetic (NOD) mice (16), a tractable model to study type 1 diabetes. Repeated injection of KRN7000 partially prevented insulitis and protected against diabetes (46, 49–51). This treatment was most effective when started early, during the initial stages of insulitis. KRN7000 was also protective when diabetes development in NOD mice was accelerated by treatment with cyclophosphamide (49), and following transplantation of freshly diabetic NOD mice with pancreatic islets (49). Repeated injection of the Th2-biasing α-GalCers OCH or C20:2 exhibited improved outcomes in the prevention of diabetes in NOD mice as compared with KRN7000 (52, 53).

### Multiple Sclerosis-Like Disease

The effects of KRN7000 and related glycolipids in the experimental autoimmune encephalomyelitis (EAE) model of multiple sclerosis-like disease in mice have been investigated using multiple myelin-derived autoantigens, mouse strains, and treatment protocols (54). Repeated subcutaneous or intraperitoneal injection of KRN7000 at the time of immunization of C57BL/6 mice with a myelin oligodendrocyte glycoprotein (MOG) peptide protected the animals against EAE (55–57). The Th2-biasing α-GalCer variant OCH was more effective than KRN7000 in protecting C57BL/6 mice against MOG-induced EAE, and was also effective when administered to mice *via* the oral route (58). In PL/J mice, treatment with KRN7000 at the time of EAE induction with myelin basic protein (MBP) also ameliorated disease (56). However, in MBP peptide-induced disease in B10.PL mice, a similar co-treatment protocol exacerbated disease, whereas KRN7000 treatment prior to disease induction was protective (56). Finally, in SJL/J mice, which contain low numbers and Th1-biased iNKT cells, KRN7000 exacerbated MBP-induced EAE (55).

### Autoimmune Arthritis

OCH but not KRN7000 was shown to protect C57BL/6 mice against collagen-induced arthritis (59). KRN7000 similarly protected against collagen-induced arthritis in DBA/1 mice when the animals were treated early but not late during the disease process (60, 61). Surprisingly, a single injection of the Th1-biasing analog α-C-GalCer was also effective in protecting DBA/1 mice against arthritis (61). KRN7000 also protected against arthritis in a model induced by immunization of DBA/1 mice with a glucose-6-phosphate isomerase peptide (62).

### Lupus-Like Autoimmunity

The impact of iNKT cell activation on both spontaneous and induced models of lupus-like autoimmunity has been explored (63). Repeated injection of KRN7000 to lupus-prone MRL/lpr mice ameliorated skin inflammation but did not affect kidney inflammation (64). In the NZB/W model of spontaneous lupus development, treatment at a young age resulted in disease amelioration (65), whereas treatment at an older age resulted in disease exacerbation (66), thereby suggesting that iNKT cell activation might have different effects when administered during different stages of the disease. Repeated KRN7000 treatment also ameliorated lupus-like disease induced by the natural hydrocarbon oil pristane in BALB/c mice, but exacerbated disease in SJL/J mice (37).

## Mechanisms of Autoimmune Modulation by iNKT Cell Antigens

**Figure 1:**
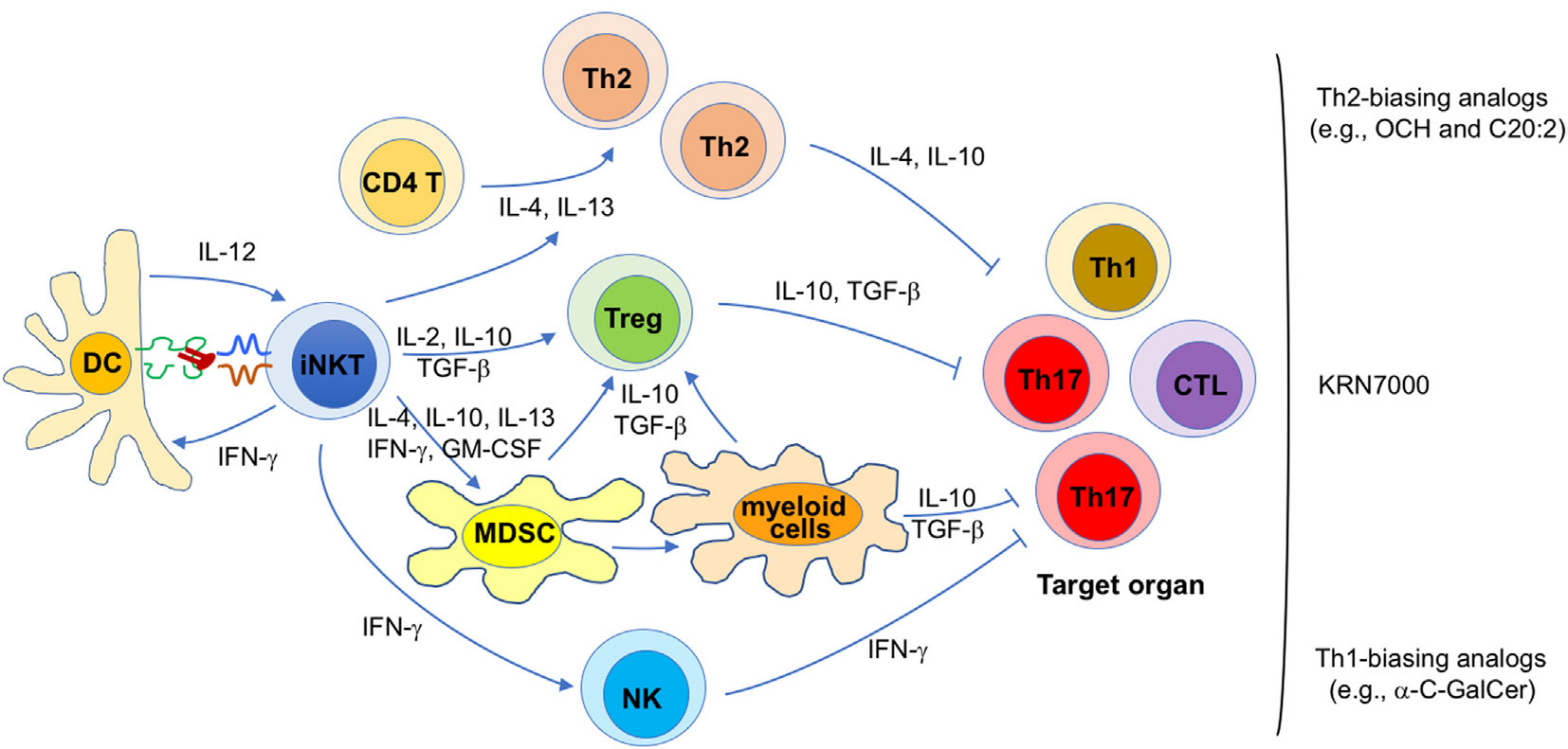
Proposed mechanisms for the therapeutic potential of invariant natural killer T (iNKT) cell antigens against autoimmunity. Diverse mechanisms that may contribute to disease protection mediated by iNKT cell antigens in different autoimmune diseases are shown. Their relative contribution is likely influenced by a variety of parameters, such as the animal model (and mouse strain) employed, various treatment variables (e.g., lipid antigen, dose, timing, frequency), and the gut microbiota. Each of these proposed mechanisms likely contributes to the efficacy of KRN7000, whereas certain mechanisms are likely more dominant for KRN7000 analogs that bias iNKT cell cytokine production profiles, as indicated.

Because the pathogenesis of autoimmune diseases is diverse, mechanisms responsible for the immunomodulatory effects of iNKT cell antigens in autoimmunity are likely diverse as well. Nevertheless, general themes by which iNKT cells exert their therapeutic activities have emerged (Figure 1).

First, disease protection afforded by KRN7000 often correlates with increased Th2 and/or reduced Th1/Th17 responses against the targeted autoantigens (37, 46, 49–51, 55–57, 60, 61, 64). Conversely, in cases where disease was exacerbated, the opposite profile was usually seen (37, 55, 56, 66). These findings suggest that a KRN7000-induced shift in the autoantigen-specific Th cell profile contributes to disease protection. This possibility is further supported by studies showing that Th2-biasing KRN7000 analogs often have superior therapeutic efficacy than the original compound (52, 53, 58, 59). Likewise, co-administration of KRN7000 with Th2-biasing anti-CD86 antibodies (67) or with the Th2-biasing cytokine IL-7 (49) also enhanced therapeutic efficacy. Such immune deviation might be imparted directly by Th2-biasing cytokines (e.g., IL-4, IL-10, and IL-13) produced by iNKT cells, or indirectly, by inducing immunoregulatory cells that subsequently promote tolerance (see below). At least in some studies, these effects of glycolipid-activated iNKT cells on autoimmune responses correlated with the capacity of the antigen to induce iNKT cell anergy (28, 29, 68, 69).

Second, KRN7000 treatment studies performed with an experimental model of autoimmune myasthenia gravis (70) and with the NOD model of diabetes (71) have shown a critical role of Foxp3^+^ Tregs in disease protection. Cytokines such as IL-2, IL-10, and TGF-β, produced by KRN7000-stimulated iNKT cells may all contribute to the generation of Foxp3^+^ Tregs in this system (47). Additionally, it is possible that induction of Foxp3^+^ Tregs involves immune suppressive myeloid lineage cells induced following iNKT cell activation (see below).

Third, there is ample evidence that KRN7000 can induce tolerogenic properties in myeloid lineage cells, including MDSCs (29, 72), DCs (50, 68, 73), macrophages (74), and neutrophils (75). MDSCs are a heterogeneous population of myeloid progenitor cells induced during inflammation by cytokines such as IL-4, IL-10, IL-13, IFN-γ, and GM-CSF (76) that can all be produced by iNKT cells. In steady-state conditions, these cells quickly differentiate into various mature myeloid cells such as macrophages, DCs and granulocytes. However, during inflammatory conditions, they rapidly expand, retain their immature phenotype, and acquire immunosuppressive properties (77). These cells employ a variety of mechanisms to suppress T cell function, including arginase-1 and inducible nitric oxide synthase activity, reactive oxygen species, immunosuppressive cytokines such as IL-10 and TGF-β, and induction of Foxp3^+^ Tregs (76, 77). The latter property of MDSCs provides a potential link between the capacity of KRN7000 to induce both MDSCs and Foxp3^+^ Tregs. Studies with the EAE model have shown a critical role of immunosuppressive MDSCs in disease protection mediated by KRN7000 (72). Similarly, a role for tolerogenic DCs was shown in EAE (68) and the NOD model of type 1 diabetes (50), and for tolerogenic M2-phenotype macrophages in EAE (74). Because MDSCs can differentiate into mature myeloid cells (76), tolerogenic DCs, and macrophages induced by KRN7000 might be derived from MDSCs (Figure 1).

Finally, in the studies with Th1-biasing KRN7000 analogs, IFN-γ was key in suppressing Th1 and Th17 responses, without promoting Th2 responses (61, 78). Although the relevant source of IFN-γ was not explored, both antigen-activated iNKT cells and transactivated NK cells produce IFN-γ under such conditions. While it remains unclear how IFN-γ might lead to suppression of pathogenic T cells, it can inhibit Th17 responses that play key roles in many autoimmune diseases (79), a possibility that is consistent with the suppressive role of iNKT cells toward Th17 responses (80). Additionally, IFN-γ might suppress pathogenic T cells by inducing anergy in them, a possibility that is supported by studies on the suppressive activities of iNKT cells against diabetogenic T cells in NOD mice (81, 82).

While each of the listed mechanisms likely contributes to the protective effects of KRN7000 on autoimmunity, immune deviation, and induction of immunosuppressive myeloid cells are likely to be most critical for the Th2-biasing KRN7000 analogs, whereas anergy induction in pathogenic T cells is likely the dominant mechanism for the Th1-biasing analogs (Figure 1).

Because these proposed mechanisms of protection involve both Th1 and Th2 cytokines, it is perhaps not surprising that IL-4, IL-10, and IFN-γ (37, 46, 49–53, 55–57, 59–61, 64, 65, 80, 83, 84), all have been shown to play a role in the tolerogenic properties of KRN7000. Even more striking, in some experimental models KRN7000 can protect against autoimmunity in an IL-4- and/or IL-10-independent manner (84, 85). Thus, multiple mechanisms might be at play that are influenced by the particular animal model of autoimmunity employed and by a variety of other factors such as the amount, timing, frequency, and route of glycolipid treatment. Another variable that likely contributes to some of the divergent findings in this field is the profound effect of the natural microbiota on the functions and therapeutic properties of iNKT cells (86).

## Future Outlook

Although KRN7000 and related iNKT cell antigens can protect against autoimmunity in many experimental models, there are also several examples of disease exacerbation. Considering the large number of variables that affect their therapeutic efficacy, a better understanding of the immunomodulatory properties of iNKT cell antigens is required, not only in mice but especially so in humans. While treatment with iNKT cell antigens in human subjects has favorable safety profiles (38), it remains uncertain if this will also be the case in patients at risk for or with autoimmunity. In particular, iNKT cells from human patients with autoimmunity are commonly reduced in numbers and often exhibit a Th1-biased cytokine production profile as compared with healthy subjects (15, 16, 18), which might be challenging to overcome during therapy. Translating the animal studies to humans is further complicated by differences in the prevalence, tissue distribution, and functions of iNKT cells between mice and humans (1–5), which likely all impact therapeutic potential. Nevertheless, the studies reviewed here have revealed cooperative interactions between immune suppressive iNKT cells, Tregs, and myeloid lineage cells, which could be exploited in combination therapies.

## Author Contributions

LK wrote the first draft, and LK and LW edited the manuscript.

## Conflict of Interest Statement

The authors declare that the research was conducted in the absence of any commercial or financial relationship that could be construed as a potential conflict of interest.
